# Supplementary material for: Assessing the knowledge and attitude towards HIV/AIDS among the general population and health care professionals in MENA region
Source: PLoS One. 2023 Jul 27;18(7):e0288838. doi: 10.1371/journal.pone.0288838 (PMC10374034; doi:10.1371/journal.pone.0288838)
Supplement: S1 File — (DOCX) [file pone.0288838.s001.docx]

**Supplementary Table 1. Overall HIV/AIDS Knowledge and Correct Responses to the HIV Questionnaire**

| **Questionnaire Topic** | **Number of participations** | **Percentage %** |
| --- | --- | --- |
| HIV is contagious | 4895 | 59·30% |
| HIV does not get transmitted through daily contact and using public bathrooms | 5092 | 61·70% |
| HIV does not transmit by contacting saliva, tears, and sweat | 3740 | 45·30% |
| HIV does not transmit through coughing and sneezing | 5086 | 61·60% |
| HIV is transmitted by blood transfusion | 7222 | 87·50% |
| HIV is sexually transmitted | 7114 | 86·20% |
| HIV does not only affect homosexuals and bisexuals. | 5755 | 69·70% |
| HIV-infected person may look healthy and feel healthy | 5974 | 72·40% |
| HIV-infected individuals develop signs of the infection quickly | 3915 | 47·40% |
| HIV patients can transmit the virus at any disease stage. | 3007 | 36·40% |
| HIV is not curable | 4205 | 50·90% |
| Is prevention the best approach to decreasing HIV incidence? | 7088 | 85·80% |

**Supplementary Table 2. Health Care Professionals' Perception of HIV/AIDS Patient Care**

| **Items** | **Responses** | **Number of HCW*** | **HCW %** |
| --- | --- | --- | --- |
| Would you provide the same quality of care to HIV-positive patients that you provide to other patients? |  |  |  |
|  | No | 355 | 13·03% |
|  | Yes | 2368 | 86·96% |
| Would you be willing to do a physical examination of a known HIV-positive patient? |  |  |  |
|  | No | 400 | 14·7% |
|  | Yes | 2323 | 85·3% |
| Do you dislike having physical contact with HIV/AIDS patients? |  |  |  |
|  | No, I am indifferent | 1675 | 61·5% |
|  | No | 156 | 5·7% |
|  | Yes | 892 | 32·8% |
| Would you interact with HIV-positive patients just like other patients? |  |  |  |
|  | No | 595 | 21·9% |
|  | Yes | 2128 | 78·1% |

**Supplementary Table 3. Attitudes of Health Care and Non-Health Care Workers Towards HIV/AIDS Patients**

|  |  | NHW | | HCW | | P^*^ |
| --- | --- | --- | --- | --- | --- | --- |
|  |  | n | % | n | % |  |
| Do you feel afraid of persons living with HIV/AIDS? | No | 2018 | 36·5% | 1532 | 56·3% | <0·001^*^ |
|  | Yes | 3515 | 63·5% | 1191 | 43·7% |  |
| Do the people that get HIV through sex or drug use have what they deserve? | No, I am indifferent | 2177 | 39·3% | 952 | 35·.0% | <0·001^*^ |
|  | No | 2277 | 41·2% | 1416 | 52·0% |  |
|  | Yes | 1079 | 19·5% | 355 | 13·0% |  |
| Is AIDS a punishment for inappropriate behavior? | No, I am indifferent | 1883 | 34·0% | 822 | 30·2% | <0·001^*^ |
|  | No | 1955 | 35·3% | 1436 | 52·7% |  |
|  | Yes | 1695 | 30·6% | 465 | 17·1% |  |
| Should HIV-positive patients be blamed for their condition? | No, I am indifferent | 1723 | 31·1% | 615 | 22·6% | <0·001^*^ |
|  | No | 3081 | 55·7% | 1890 | 69·4% |  |
|  | Yes | 729 | 13·2% | 218 | 8·0% |  |
| Would you feel ashamed if someone you know got HIV? | No | 3749 | 67·8% | 2028 | 74·5% | <0·001^*^ |
|  | Yes | 1784 | 32·2% | 695 | 25·5% |  |
| Will your attitude change towards your colleagues if they contract HIV? | No | 3523 | 63·7% | 2141 | 78·6% | <0·001^*^ |
|  | Yes | 2010 | 36·3% | 582 | 21·4% |  |
| Will you spend time with your friend if you learn that he/she has HIV? | No | 2114 | 38·2% | 595 | 21·9% | <0·001^*^ |
|  | Yes | 3419 | 61·8% | 2128 | 78·1% |  |
| Do you approve of the marriage of an HIV-positive individual to an HIV-negative individual? | No | 4900 | 88·6% | 2338 | 85·9% | <0·001^*^ |
|  | Yes | 633 | 11·4% | 385 | 14·1% |  |
| Do you approve of the marriage of two HIV-positive individuals? | No | 2683 | 48·5% | 1099 | 40·4% | <0·001^*^ |
|  | Yes | 2850 | 51·5% | 1624 | 59·6% |  |
| Do you agree with obligatory pre-marital HIV testing as a precautionary measure | No | 238 | 4·3% | 103 | 3·8% | 0·265 |
|  | Yes | 5295 | 95·7% | 2620 | 96·2% |  |

Note: HCW=Health Care Worker, NHW=Non-Health Care Worker, ^*^Chi-square test

**Supplementary Table 4. Comparison of Mean Knowledge Score of HIV/AIDS Among Different Groups**

| **Variables** | | **Mean ± SD** | **KW-H** | **df** | **p*** |
| --- | --- | --- | --- | --- | --- |
| **Age**  **(Years)** | 15-19 | 9·23±3·72^b^ | 36·156 | 4 | <0·001 |
|  | 20-29 | 9·89±3·95^a^ |  |  |  |
|  | 30-39 | 9·66±3·88^a^ |  |  |  |
|  | 40-49 | 9·66±3·69^a^ |  |  |  |
|  | Above 50 | 9·32±3·86**^b^** |  |  |  |
| **Nationality** | Saudi Arabian | 9·47±3·85**^c^** | 104·431 | 3 | <0·001 |
|  | GCC | 10·11±3·83**^b^** |  |  |  |
|  | MENA | 9·90±3·93**^b^** |  |  |  |
|  | Others | 11·12±3·65**^a^** |  |  |  |
| **Region** | Central | 9.36±3.87 | 9·362 | 4 | 0·053 |
|  | Eastern | 9·66±3·82 |  |  |  |
|  | Northern | 9·44±3·65 |  |  |  |
|  | Western | 9·76±3·91 |  |  |  |
|  | Southern | 9·57±3·61 |  |  |  |
| **Marital Status** | Single | 9·79±3·91**^a^** | 15·266 | 2 | <0·001 |
|  | Married | 9·52±3·83**^b^** |  |  |  |
|  | Previously married | 9·30±3·72**^c^** |  |  |  |
| **Education** | Primary | 8.54±3.68^d^ | 168·306 | 3 | <0·001 |
|  | Secondary | 9·18±3·87^cbd^ |  |  |  |
|  | Under-Graduate | 9·41±3·85^b^ |  |  |  |
|  | Post-Graduate | 10·79±3·69^a^ |  |  |  |
| **Income**  **(in dollars)** | Less than 1000 | 9·60±3·90^a^ | 12·803 | 3 | 0·005 |
|  | 1000-4999 | 9·51±3·87^a^ |  |  |  |
|  | 5000-10000 | 9·84±3·84^a^ |  |  |  |
|  | More than 10000 | 9·98±3·72^a^ |  |  |  |
| **Occupation** | Student | 9·75±3v87 | 4·952 | 3 | 0·175 |
|  | Employed | 9·58±3·87 |  |  |  |
|  | Self-employed | 9·70±3·70 |  |  |  |
|  | Unemployed | 9·84±3·99 |  |  |  |

Note: KW-H=Kruskal-Wallis H, df=degrees of freedom, *ruskal-wallis test,

Note: Different letters (a,b,c,d) in the same column indicate significant differences between groups (p<0.05), a same letter in the single column indicates no significant differences (p>0.05)

**Supplementary Table 5. Overall HIV/AIDS Knowledge and Stigma Score per Group**

| Questions | Responses | Gender | | | Marital Status | | | | Education | | | | | Income (SAR) | | | | |
| --- | --- | --- | --- | --- | --- | --- | --- | --- | --- | --- | --- | --- | --- | --- | --- | --- | --- | --- |
|  |  | Male | Female | P | Single | Married | Previously married | P | Primary | Secondary | UG | PG | P | <1000 | 1000-4999 | 5000-10000 | > 10000 | P |
|  |  | % | % |  | % | % | % |  | % | % | % | % |  | % | % | % | % |  |
| Q14 | Incorrect | 38·7 | 41·9 | ·004* | 38·8 | 42·3 | 45·9 | ·001* | 48·2 | 43·2 | 42·3 | 31·8 | ·000* | 39·3 | 43·2 | 39·6 | 39·3 | ·009* |
|  | Correct | 61·3 | 58·1 |  | 61·2 | 57·7 | 54·1 |  | 51·8 | 56·8 | 57·7 | 68·2 |  | 60·7 | 56·8 | 60·4 | 60·7 |  |
| Q15 | Incorrect | 36·0 | 39·7 | ·001* | 37·8 | 38·8 | 39·8 | 0·578 | 48·2 | 42·9 | 40·9 | 28·2 | ·000* | 39·2 | 39·6 | 36·2 | 34·2 | ·007* |
|  | Correct | 64.0 | 60.3 |  | 62.2 | 61.2 | 60.2 |  | 51·8 | 57.1 | 59.1 | 71.8 |  | 60.8 | 60.4 | 63.8 | 65.8 |  |
| Q16 | Incorrect | 51.6 | 56.5 | .000* | 53.9 | 55.6 | 54.1 | 0.299 | 63·3 | 58.3 | 56.5 | 50.0 | .000* | 55.7 | 54.3 | 52.9 | 55.2 | 0.377 |
|  | Correct | 48.4 | 43.5 |  | 46.1 | 44.4 | 45.9 |  | 36·7 | 41.7 | 43.5 | 50.0 |  | 44.3 | 45.7 | 47.1 | 44.8 |  |
| Q17 | Incorrect | 36.4 | 39.6 | .004* | 37.9 | 38.8 | 40.1 | 0.599 | 48·7 | 45.1 | 40.5 | 27.0 | .000* | 39.7 | 39.2 | 35.8 | 35.2 | .015* |
|  | Correct | 63.6 | 60.4 |  | 62.1 | 61.2 | 59.9 |  | 51.3 | 54.9 | 59.5 | 73.0 |  | 60.3 | 60.8 | 64.2 | 64.8 |  |
| Q18 | Incorrect | 11.0 | 13.4 | .002* | 12.1 | 12.9 | 14.0 | 0.410 | 17.1 | 15.5 | 13.3 | 7.8 | .000* | 12.8 | 13.3 | 11.2 | 11.0 | 0.116 |
|  | Correct | 89.0 | 86.6 |  | 87.9 | 87.1 | 86.0 |  | 82.9 | 84.5 | 86.7 | 92.2 |  | 87.2 | 86.7 | 88.8 | 89.0 |  |
| Q19 | Incorrect | 12.8 | 14.4 | .045* | 13.1 | 14.4 | 16.8 | 0.071 | 20.6 | 16.6 | 14.4 | 8.8 | .000* | 13.4 | 15.4 | 12.7 | 11.9 | .013* |
|  | Correct | 87.2 | 85.6 |  | 86.9 | 85.6 | 83.2 |  | 79.4 | 83.4 | 85.6 | 91.2 |  | 86.6 | 84.6 | 87.3 | 88.1 |  |
| Q20 | Incorrect | 29.9 | 30.5 | 0.574 | 28.6 | 31.7 | 36.0 | .001* | 38.2 | 31.9 | 30.1 | 30.9 | 0.075 | 29.5 | 30.9 | 29.9 | 31.6 | 0.525 |
|  | Correct | 70.1 | 69.5 |  | 71.4 | 68.3 | 64.0 |  | 61.8 | 68.1 | 69.9 | 69.1 |  | 70.5 | 69.1 | 70.1 | 68.4 |  |
| Q21 | Incorrect | 25.7 | 28.7 | .003* | 26.7 | 28.5 | 29.1 | 0.158 | 39.2 | 32.4 | 29.1 | 19.2 | .000* | 27.9 | 28.3 | 28.1 | 24.2 | 0.092 |
|  | Correct | 74.3 | 71.3 |  | 73.3 | 71.5 | 70.9 |  | 60.8 | 67.6 | 70.9 | 80.8 |  | 72.1 | 71.7 | 71.9 | 75.8 |  |
| Q22 | Incorrect | 50.6 | 53.7 | .007* | 51.8 | 53.0 | 56.9 | 0.134 | 61.8 | 58.9 | 54.3 | 41.7 | .000* | 52.4 | 54.5 | 51.6 | 48.6 | .014* |
|  | Correct | 49.4 | 46.3 |  | 48.2 | 47.0 | 43.1 |  | 38.2 | 41.1 | 45.7 | 58.3 |  | 47.6 | 45.5 | 48.4 | 51.4 |  |
| Q23 | Incorrect | 61.3 | 64.9 | .001* | 62.7 | 64.1 | 68.7 | .048* | 67.3 | 65.2 | 64.5 | 54.1 | .000* | 63.2 | 64.3 | 64.3 | 61.6 | 0.434 |
|  | Correct | 38.7 | 35.1 |  | 37.3 | 35.9 | 31.3 |  | 32.7 | 34.8 | 35.5 | 45.9 |  | 36.8 | 35.7 | 35.7 | 38.4 |  |
| Q24 | Incorrect | 51.7 | 47.6 | .000* | 46.4 | 51.5 | 54.7 | .000* | 53.8 | 50.6 | 50.3 | 43.2 | .000* | 47.1 | 51.2 | 49.3 | 48.9 | .017* |
|  | Correct | 48.3 | 52.4 |  | 53.6 | 48.5 | 45.3 |  | 46.2 | 49.4 | 49.7 | 56.8 |  | 52.9 | 48.8 | 50.7 | 51.1 |  |
| Q25 | Incorrect | 13.1 | 14.8 | .038* | 15.6 | 12.5 | 14.3 | .000* | 16.6 | 14.7 | 15.1 | 12.1 | .048* | 15.3 | 14.4 | 13.4 | 10.4 | .002* |
|  | Correct | 86.9 | 85.2 |  | 84.4 | 87.5 | 85.7 |  | 83.4 | 85.3 | 84.9 | 87.9 |  | 84.7 | 85.6 | 86.6 | 89.6 |  |
| Q44 | Incorrect | 50.0 | 50.6 | 0.599 | 49.8 | 51.0 | 51.1 | 0.548 | 51.3 | 53.4 | 52.2 | 41.9 | .000* | 50.5 | 51.1 | 50.4 | 47.5 | 0.300 |
|  | Correct | 50.0 | 49.4 |  | 50.2 | 49.0 | 48.9 |  | 48.7 | 46.6 | 47.8 | 58.1 |  | 49.5 | 48.9 | 49.6 | 52.5 |  |
| Q45 | Incorrect | 62.9 | 68.6 | .000* | 65.4 | 68.0 | 64.8 | .040* | 75.4 | 66.1 | 67.9 | 60.7 | .000* | 68.3 | 66.9 | 63.9 | 62.8 | .002* |
|  | Correct | 37.1 | 31.4 |  | 34.6 | 32.0 | 35.2 |  | 24.6 | 33.9 | 32.1 | 39.3 |  | 31.7 | 33.1 | 36.1 | 37.2 |  |
| Q46 | Incorrect | 51.6 | 51.5 | 0.970 | 51.4 | 51.8 | 49.7 | 0.739 | 54.3 | 55.0 | 53.5 | 43.7 | .000* | 52.6 | 51.7 | 49.4 | 50.2 | 21.5 |
|  | Correct | 48.4 | 48.5 |  | 48.6 | 48.2 | 50.3 |  | 45.7 | 45.0 | 46.5 | 56.3 |  | 47.4 | 48.3 | 50.6 | 49.8 |  |
| Q47 | Incorrect | 57.2 | 61.4 | .000* | 58.7 | 61.0 | 61.8 | 0.080 | 67.8 | 61.1 | 62.5 | 51.2 | .000* | 62.0 | 59.1 | 58.8 | 56.3 | .006* |
|  | Correct | 42.8 | 38.6 |  | 41.3 | 39.0 | 38.2 |  | 32.2 | 38.9 | 37.5 | 48.8 |  | 38.0 | 40.9 | 41.2 | 43.7 |  |
| Q48 | Incorrect | 69.3 | 72.1 | .006* | 69.8 | 72.4 | 72.5 | .031* | 73.9 | 70.7 | 71.7 | 68.4 | 0.106 | 70.9 | 71.6 | 68.9 | 72.9 | 0.180 |
|  | Correct | 30.7 | 27.9 |  | 30.2 | 27.6 | 27.5 |  | 26.1 | 29.3 | 28.3 | 31.6 |  | 29.1 | 28.4 | 31.1 | 27.1 |  |

Note: UG=Under-Graduate, PG=Post-Graduate.

**Supplementary Table 6. HIV/AIDS Knowledge Through Media as a Source of Information**

| **Variables** | | **Mean ± SD** | **KW-H** | **df** | **p*** |
| --- | --- | --- | --- | --- | --- |
| **Mass media Source** | Almost every day | 9·62±3.86 | 3·474 | 3 | 0·324 |
|  | At least once a week | 9·61±3·81 |  |  |  |
|  | At least less than once a week | 9·81±3·93 |  |  |  |
|  | Not at all | 9·61±3.89 |  |  |  |
| **Social media** | Almost every day | 9·72±3·87^a^ | 14·434 | 3 | 0·002 |
|  | At least once a week | 9·45±3·81^a^ |  |  |  |
|  | At least less than once a week | 9·19±3·95^a^ |  |  |  |
|  | Not at all | 9·33±3·83^a^ |  |  |  |
