# Supplementary material for: Assessing the knowledge and attitude towards HIV/AIDS among the general population and health care professionals in MENA region
Source: PLoS One. 2023 Jul 27;18(7):e0288838. doi: 10.1371/journal.pone.0288838 (PMC10374034; doi:10.1371/journal.pone.0288838)
Supplement: Attachment — Submitted filename: Reviewer-PLOS ONE.docx [file pone.0288838.s003.docx]

**Dear Editor in Chief	March 25, 2023**

PLOS ONE

We would like to thank the reviewers and the Editorial Office again for their time and their critical review of our manuscript. We are pleased to inform you that all the comments raised by the reviewers were taken carefully responded point-by-point. Please find below our point-by-point responses. All the changes in the attached revised manuscript are indicated in track change.

We thank you again for the opportunity to resubmit our paper.

Yours faithfully

Maha Al-Mozaini, PhD

**Reviewer comments:**

1. You indicated that you had ethical approval for your study. In your Methods section, please ensure you have also stated whether you obtained consent from parents or guardians of the minors included in the study or whether the research ethics committee or IRB specifically waived the need for their consent.

**Response**: we have included this statement at the Ethics part of the manuscript.

2. Please update the Figures captions, all figures’ captions as Figure 1(Figure 1. HIV Knowledge Score Among Gender and HCW VS NHW, Figure 1. Frequency of Utilization of Mass Media and social media among the Study Participants)

**Response: amended**

**Reviewer comments**

**1. Is the manuscript technically sound, and do the data support the conclusions?**

The manuscript must describe a technically sound piece of scientific research with data that supports the conclusions. Experiments must have been conducted rigorously, with appropriate controls, replication, and sample sizes. The conclusions must be drawn appropriately based on the data presented.

Reviewer #1: Yes

Reviewer #2: Partly

**Response 1**: On behalf of all the coauthors and myself, we appreciate this positive feedback.

**2. Has the statistical analysis been performed appropriately and rigorously?**

Reviewer #1: Yes

Reviewer #2: N/A

**Response 2**: We appreciate the reviewers positive support, we did indeed had an expert biostatistician analyzing our data.

**3. Have the authors made all data underlying the findings in their manuscript fully available?**

Reviewer #1: Yes

Reviewer #2: Yes

**Response 3:** Indeed, all the data are available in the supplementary files.

**4. Is the manuscript presented in an intelligible fashion and written in standard English?**

Reviewer #1: Yes

Reviewer #2: Yes

**Response 4**: Once again on behalf of the co-authors and myself we are grateful and appreciate the positive feedback.
 **5. Review Comments to the Author

Reviewer #1:** This is a very important manuscript. It highlights the general public knowledge on HIV/AIDS. most importantly its highlight the perception of health workers towards HIV patients. The attitude of healthcare workers needs more improvement to provide the best amount of care to the HIV-afflicted.

**Response 4 for Reviewer 1**: Indeed these data and our finding once published will share it with policy makers and the ministry of health to start awareness campaigns in the country especially among health care workers in order to provide the best healthcare to this group of patients without discrimination.

**Reviewer #2:** This is a good study, unfortunately dataset and results are not unique. The paper has many shortcomings in regard to data analyses and text. In my opinion, However, it needs to more clarify regarding to the protocol of sample collection and protocol which is used to online survey. In addition, results has not been used to its full text. I think need more in-depth analysis of the data. I also suggest siting more relevant and new literatures.

**Response 4 for Reviewer 2**: Valid point and accordingly, we have addressed this in the data collection method by describing how the online survey was disseminated through social media posts such as the twitter pots of our institute KFSHRC and WhatsApp platform of data collection volunteers.

On behalf of the co-authors and myself we are grateful and appreciate your time in reviewing our project and we hope to collaborate on many more projects in the near future.

Thank you
