# Supplementary material for: Assessing the knowledge and attitude towards HIV/AIDS among the general population and health care professionals in MENA region
Source: PLoS One. 2023 Jul 27;18(7):e0288838. doi: 10.1371/journal.pone.0288838 (PMC10374034; doi:10.1371/journal.pone.0288838)
Supplement: Attachment — Submitted filename: Reviewer-PLOS ONE.docx [file pone.0288838.s004.docx]

**Dear Editor in Chief	June 26, 2023**

PLOS ONE

We would like to thank again the reviewers and the Editorial Office for their time and their critical review of our manuscript and the acceptance of our manuscript with minor revision. We are pleased to inform you that all the comments raised by the reviewers were taken carefully and responded point-by-point. Please find below our point-by-point responses. All the changes in the attached revised manuscript are indicated in track change.

We thank you again for the opportunity.

Yours faithfully

Maha Al-Mozaini, PhD

**Reviewer comments:**

1. There is discrepancy in the statistical tool used to analysed in the data. In the abstract, R-statistical tool was mentioned, while SPSS was mentioned in the methods section of the manuscript. The author needs to address it.

**Response:** We thank the reviewers for noticing this minor mistake, we have corrected this by adding the R statistical tool that was utilized for our study. Red highlight in the manuscript in the statistical methodology section.

2. Some participants were less than 18 years. The consent of their parents/guardians are required before they can be interviewed. The authors were silent on this issue. The authors need to address this issue.

**Response**: On behalf of all the coauthors and myself, we appreciate this point, accordingly we have addressed this in the ethics section that is highlighted in red. This comment was added ‘Some participants were less than 18 years, 12% of our study participant. The consent of their parents/guardians were obtained before they were interviewed’.

On behalf of the co-authors and myself we are grateful and appreciate your time in reviewing our project and we hope to collaborate on many more projects soon.

Thank you
